# Clinical Outcomes After a Digital Musculoskeletal Program for Acute and Subacute Pain: Observational, Longitudinal Study With Comparison Group

**DOI:** 10.2196/38214

**Published:** 2022-06-27

**Authors:** Grace Wang, Manshu Yang, Mindy Hong, Jeffrey Krauss, Jeannie F Bailey

**Affiliations:** 1 Hinge Health, Inc San Francisco, CA United States; 2 Department of Psychology University of Rhode Island Kingston, RI United States; 3 Department of Orthopaedic Surgery University of California, San Francisco San Francisco, CA United States

**Keywords:** telemedicine, acute, subacute, musculoskeletal, pain, function, clinical, quality of life, intervention, longitudinal study, physical therapy, physiotherapy, physical therapist, physiotherapist, exercise, physical activity, telehealth, eHealth, digital health, patient education, education material, education resource, health resource, mHealth, mobile health, health app, observational study, video consult, eConsult, virtual care

## Abstract

**Background:**

Telerehabilitation for musculoskeletal (MSK) conditions may produce similar or better outcomes than usual care, but most telerehabilitation studies address only chronic or postsurgical pain.

**Objective:**

We aimed to examine pain and function at 3, 6, and 12 weeks for individuals with acute and subacute MSK pain who took part in a digital MSK program versus a nonparticipant comparison group.

**Methods:**

We conducted an observational, longitudinal study with a nonparticipant comparison group. The intervention group had video visits with physical therapists who recommended exercise therapies and educational articles delivered via an app. Nonparticipants were those who were registered but unable to participate because their benefit coverage had not yet begun. We collected pain and function outcomes through surveys delivered at 3-, 6-, and 12-week follow-ups. We conducted descriptive analyses, unadjusted regression, and mixed effects regression adjusting for baseline characteristics, time as fixed effects, and a time*group interaction term.

**Results:**

The analysis included data from 675 nonparticipants and 262 intervention group participants. Compared to baseline, the intervention group showed significantly more pain improvement at 3, 6, and 12 weeks versus nonparticipants after adjusting for baseline factors. Specifically, the intervention group’s pain scores decreased by 55.8% at 3 weeks versus baseline, 69.1% at 6 weeks, and 73% at 12 weeks. The intervention group’s adjusted pain scores decreased from 43.7 (95% CI 41.1-46.2) at baseline to 19.3 (95% CI 16.8-21.8) at 3 weeks to 13.5 (95% CI 10.8-16.2) at 6 weeks to 11.8 (95% CI 9-14.6) at 12 weeks. In contrast, nonparticipants’ pain scores decreased by 30.8% at 3 weeks versus baseline, 45.8% at 6 weeks, and 46.7% at 12 weeks. Nonparticipants’ adjusted pain scores decreased from 43.8 (95% CI 42-45.5) at baseline to 30.3 (95% CI 27.1-33.5) at 3 weeks to 23.7 (95% CI 20-27.5) at 6 weeks to 23.3 (95% CI 19.6-27) at 12 weeks. After adjustments, the percentage of participants reporting that pain was better or much better at follow-up was significantly higher by 40.6% at 3 weeks, 31.4% at 6 weeks, and 31.2% at 12 weeks for intervention group participants versus nonparticipants. After adjustments, the percentage of participants with meaningful functional improvement at follow-up was significantly higher by 15.2% at 3 weeks and 24.6% at 12 weeks for intervention group participants versus nonparticipants.

**Conclusions:**

A digital MSK program may help to improve pain and function in the short term among those with acute and subacute MSK pain.

## Introduction

Acute, subacute, and chronic musculoskeletal (MSK) conditions are a leading cause of disability and cost in the United States [1]. The rates of back pain, neck pain, and other MSK disorders in the United States are among the highest in the world [1]. In 2019, 39% of American adults reported back pain, 37% reported lower limb pain (eg, hips, knees, and feet), and 31% reported upper limb pain (eg, hands, arms, and shoulders) in the 3 months prior [2].

MSK conditions include injuries or pain in joints, ligaments, muscles, nerves, tendons, and structures that support limbs, neck, and back. They may be a result of exertion, repetitive motions, strain, or exposure to force, vibration, or awkward posture [3]. Acute pain is often defined as lasting 4 weeks or less. Subacute pain duration is from 4 to 12 weeks, and chronic pain duration is more than 12 weeks [4,5].

MSK conditions are a common cause of health care use in the United States. For example, 72.4 million office visits and 9.9 million emergency department visits were for MSK conditions in 2018 [6,7]. Of these, more than 4 million emergency department visits were for sprains and strains alone. Although providers and patients may pursue different pain management approaches for acute and subacute needs, numerous studies and clinical guidelines recommend education and exercise [8,9].

Telerehabilitation, a branch of telehealth that uses telecommunications technologies to control or monitor remote rehabilitation, is increasingly used to deliver MSK care [10]. Telerehabilitation for MSK conditions may produce similar or even better pain-, functional-, and health-related quality of life outcomes than usual care, but most telerehabilitation studies address only chronic or postsurgical pain [10-12]. Therefore, we aimed to determine whether telerehabilitation was associated with improved clinical outcomes in acute and subacute MSK conditions. Our primary objective was to examine pain and function at 3, 6, and 12 weeks for participants of a digital acute MSK program versus a nonparticipant comparison group. A secondary objective was to examine engagement among the intervention group. The findings contribute to a growing evidence base about the role of digital health for managing a range of MSK needs.

## Methods

### Study Design

We conducted an observational, prospective cohort study comparing digital MSK acute program participants (herein, intervention group) to nonparticipants at 3, 6, and 12 weeks.

### Acute Program

Employers offered the acute program to employees and adult dependents as a health benefit. Recruitment was conducted through post and email. Registration involved creating a member profile and completing an application over the internet.

Developed by physical therapists (PTs), the acute program’s goal was to help participants address acute or subacute MSK pain through digital physical therapy consultation, exercise therapy, and education. Participants had access to an acute program app for use on personal tablets or smartphones.

The acute program began with a video visit with a licensed PT. The PT conducted a subjective interview to learn more about the participant’s history and goals and guided them through a series of movement tests to assess their current level of function. After the video visit, the PT provided a plan with recommended exercises and education that were available to participants through the app. The app provided this information through “sessions.”

Each session presented a set of exercises that were specific to acute back, knee, shoulder, hip, neck/upper back, elbow/wrist/hand, or ankle/foot pain. Each session included stretching, strengthening, balancing, and mobility activities, based on the participant’s functional limitations and goals determined during the consultation. The session presented 1 to 2 sets of 3 to 10 repetitions of each exercise (depending on the difficulty and type of exercise), with each session’s duration ranging from 5 to 20 minutes. Graphics along with written and audio cues demonstrated how to perform the exercises, the number of repetitions for each exercise, and how long to hold the positions. As participants progressed through the program, their exercises were adjusted by the PT to gradually advance them toward their goals. This included adjusting the exercise variation, number of repetitions, hold time, and use of resistance with resistance bands (if applicable).

After participants completed the exercises for that session, the app presented educational resources about acute and subacute MSK pain–related topics, such as pain neuroscience, movement, treatment options, coping techniques, healthy lifestyle practices, relaxation tools, social support, and habit formation. Lastly, the participant was able to leave a note for their PT, rate their pain, or record any additional activity they had completed recently. As a wholly digital program, participants could choose when and where to meet with PTs via video and complete sessions.

### Study Participants

First, for each week between July and October 2021, we identified individuals meeting the inclusion and exclusion criteria based on information provided in the application. Inclusion criteria were aged ≥18 years; back, knee, shoulder, hip, or neck pain; visual analog scale (VAS) pain score >0; pain for less than 12 weeks; and covered by employer’s health plan. Exclusion criteria were signs of fracture, joint instability, infection, cancer, and cauda equina syndrome.

Second, we categorized the individuals as part of the intervention or nonparticipant group. The intervention group had a first video visit with a PT in the past week and a published care plan. Nonparticipants were those who applied to the acute program but were declined because their employers did not yet offer the acute program as a benefit. Everyone in the intervention group and a sample of the nonparticipants were invited to the study. To sample nonparticipants, we stratified them by pain region (ie, back, knee, shoulder, hip, and neck) and conducted a propensity score match based on baseline pain and function.

Between August and November 2021, we invited participants to complete an email survey 3 weeks after registration (nonparticipants) or video visit (intervention). We excluded individuals who did not provide informed consent or those who had pain for more than 12 weeks. Between August 2021 and January 2022, we sent surveys at 6 and 12 weeks after registration (nonparticipants) or video visit (intervention) to those who completed the 3-week follow-up survey and agreed to be recontacted (Table 1).

**Table 1:** Timeline for an example cohort who registered or had video visits between July 7, 2021, and July 13, 2021.

Date	Event
July 7-13	Nonparticipant group registers Intervention group has a physical therapist video visit
July 14	Apply inclusion and exclusion criteria and sample
August 4-11	Complete 3-week follow-up by email survey
August 25 to September 1	Complete 6-week follow-up by email survey
October 6-13	Complete 12-week follow-up by email survey

### Ethics Approval

Study subjects acknowledged via the internet that they provided informed consent. The WIRB-Copernicus Group Institutional Review Board (Office of Human Research Protections/Food and Drug Administration Institutional Review Board registration number IRB00000533) at the WIRB-Copernicus Group reviewed and approved this study.

### Outcomes

The primary outcome was pain improvement based on the response to the following question: “Over the past 24 hours, how bad was your [back/knee/shoulder/hip/neck] pain?” with a score from 0 (none) to 100 (worst imaginable).

A secondary outcome was the patient’s global impression of change (PGIC) based on the response to the following question: “Compared to when you first registered for Hinge Health, how would you rate your [back/knee/shoulder/hip/neck] pain now?” Pain rated as better or much better was coded as 1; pain rated as much worse, worse, a little worse, unchanged, or a little better was coded as 0.

Another secondary outcome was minimal clinically important difference (MCID) in functional improvement (herein, functional improvement). To create this dichotomous variable (no/yes), we gathered responses to the 11-item Roland Morris Disability Questionnaire (RMDQ-11, back only), Knee injury and Osteoarthritis Outcome Score Physical Function Short form (KOOS-PS, knee only), Hip disability and Osteoarthritis Outcome Score Physical Function Short form (HOOS-PS, hip only), Shoulder Pain and Disability Index (SPADI, shoulder only), and Neck Pain and Disability Scale short form (sf-NPAD, neck only). Next, we calculated the change from baseline to follow-up. MCID in functional improvement is defined as either at least 30% improvement on the RMDQ-11 [13,14]; 8-point improvement on the KOOS-PS [15-17]; 9.3-point improvement on the HOOS-PS [18,19]; 13-point improvement on the SPADI [20-22]; 12-point improvement on the sf-NPAD [23,24]; or no limitations at follow-up.

For the intervention group’s engagement, we collected the number of video visits and app-based exercise therapy sessions completed by 12 weeks. Exercise completion was recorded when participants used the app. We did not record exercises completed outside the app.

### Exposures

Nonparticipants were those who were registered but did not take part in the acute program. The intervention group had one or more PT video visits, a published care plan, and access to exercise guidance and education via the acute program app.

### Confounders

Model covariates included registration month (July, August, September, or October), age at baseline, pain region (back, knee, shoulder, hip, or neck), and the use of health care services at 12 weeks (no/yes). The health care services were conservative care (eg, office visit with a doctor or physical therapist), over-the-counter medications, prescription pain medications, and invasive procedures (eg, emergency department or urgent care center visit, overnight stay in a hospital, injections, or surgery).

### Data Sources

The web-based application completed at program registration provided baseline data. We emailed follow-up surveys and up to 2 reminders at 3, 6, and 12 weeks after registration (nonparticipants) or the first PT video visit (intervention). Respondents received gift cards for US $20 at 3 weeks, US $25 at 6 weeks, and US $35 at 12 weeks.

### Study Size

Sample size was based on detecting noninferiority of the intervention versus nonparticipants at 6 weeks after registering or video visit. For VAS pain scores, we chose a noninferiority margin of 10 points because this is less than the 20-point reduction for MCID in pain improvement [25]. Assuming SDs of 21.4 for pain [26], 80% power, and a 1-sided 2.5% significance level, we needed 57 participants per arm (N=114).

### Statistical Methods

Summary statistics were estimated for baseline characteristics of age, pain region, registration month, and baseline pain. We conducted 2-tailed *t* tests (for continuous variables) and chi-square tests (for categorical variables) to show whether there were significant differences between the intervention group and nonparticipants at baseline. Descriptive statistics reported at 3, 6, and 12 weeks were mean (SD) VAS pain scores, the number and percentage of participants who perceived better or much better pain (PGIC) at follow-up compared to registration, and the number and percentage of participants who achieved an MCID in functional improvement.

Unadjusted and adjusted linear mixed effects regression models were used to model pain improvement, and generalized linear mixed effects models were used for PGIC and functional improvement. Covariates were baseline age, pain region, registration month, and health care service use at 12 weeks. PGIC and functional improvement models also included baseline pain. Time was treated as a categorical predictor to allow the modeling of nonlinear change trends over time. A 2-way time*group interaction term captured the treatment effect at each time point. Estimated predicted probabilities and marginal effects are presented below.

The primary analysis used all available data. The maximum likelihood estimation method was used, assuming data were missing at random. Analyses were performed in Stata (version 17.0; StataCorp) and R statistical software (version 4.0.5; R Foundation for Statistical Computing).

## Results

### Flowchart

**Figure 1:**
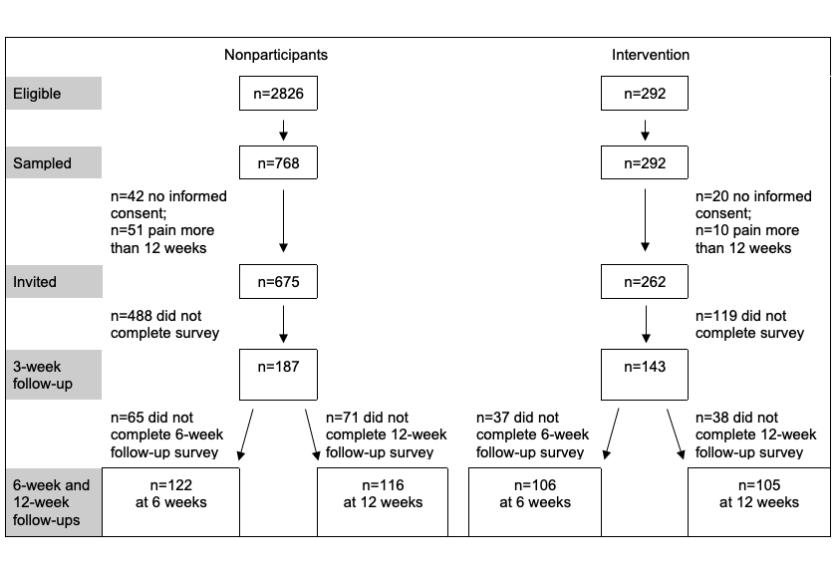
Flowchart, by group.

Figure 1 reports the intervention and nonparticipant groups at each study stage.

### Sample Characteristics

Table 2 shows the baseline characteristics for the nonparticipant and intervention groups. We detected no significant differences between the 2 groups at baseline. The mean age of the total sample was 44.1 (SD 11.9) years. At registration, mean pain was 43.0 (SD 22.3) out of 100. The largest (31.9%, 299/937) percentage of the sample registered for back pain and the smallest (13.8%, 129/937) registered for hip pain.

**Table 2:** Baseline characteristics.

Characteristic			Nonparticipant group (n=675)		Intervention group (n=262)		All participants (N=937)
Age (year), mean (SD)			44.0 (12.1)		44.4 (11.3)		44.1 (11.9)
Baseline pain, mean (SD)			42.9 (22.5)		43.2 (21.7)		43.0 (22.3)
**Pain region, n (%)**							
	Back	225 (33.3)		74 (28.2)		299 (31.9)	
	Hip	87 (12.9)		42 (16)		129 (13.8)	
	Knee	119 (17.6)		53 (20.2)		172 (18.4)	
	Neck	140 (20.7)		49 (18.7)		189 (20.2)	
	Shoulder	104 (15.4)		44 (16.8)		148 (15.8)	
**Registration month, n (%)**							
	July	124 (18.4)		54 (20.6)		178 (19)	
	August	170 (25.2)		60 (22.9)		230 (24.5)	
	September	236 (35)		77 (29.4)		313 (33.4)	
	October	145 (21.5)		71 (27.1)		216 (23.1)	

### Descriptive Results

Nonparticipants’ absolute decrease in pain from baseline was 11.5 points at 3 weeks, 17.9 points at 6 weeks, and 18.2 points at 12 weeks. The intervention group’s absolute decrease in pain from baseline was 24.0 points at 3 weeks, 29.0 points at 6 weeks, and 30.5 points at 12 weeks (Table 3).

The percentage of participants reporting that pain as better or much better (PGIC) was 69.3% (104/150) at 3 weeks, 73.9% (85/115) at 6 weeks, and 78.5% (95/121) at 12 weeks in the intervention group. For nonparticipants, the percentages were 26% (51/196) at 3 weeks, 38.5% (50/130) at 6 weeks, and 43.1% (53/123) at 12 weeks. PGIC was higher for the intervention group than the nonparticipant group by 43.3 percentage points at 3 weeks, 35.4 percentage points at 6 weeks, and 35.5 percentage points at 12 weeks.

The percentage of participants reporting meaningful functional improvement was 56.5% (105/186) at 3 weeks, 67.9% (91/134) at 6 weeks, and 77.7% (94/121) at 12 weeks in the intervention group. For nonparticipants, the percentages were 39.3% (77/196) at 3 weeks, 51.6% (66/128) at 6 weeks, and 50.8% (62/122) at 12 weeks. The percentage reporting functional improvement was higher for the intervention group than the nonparticipant group by 17.2 percentage points at 3 weeks, 16.3 percentage points at 6 weeks, and 26.9 percentage points at 12 weeks (Table 3).

**Table 3:** Descriptive results: outcomes over time for nonparticipant and intervention groups.

Outcome, timepoint		Nonparticipant group	Intervention group
**Pain score, mean (SD)**			
	Baseline	42.9 (22.5)	43.2 (21.7)
	3 weeks	31.4 (22.8)	19.2 (17.9)
	6 weeks	25.0 (21.6)	14.2 (16.0)
	12 weeks	24.7 (20.5)	12.7 (14.2)
**Patient’s global impression of change, n (%)**			
	3 weeks (nonparticipant group: n=196; intervention group: n=150)	51 (26)	104 (69.3)
	6 weeks (nonparticipant group: n=130; intervention group: n=115)	50 (38.5)	85 (73.9)
	12 weeks (nonparticipant group: n=123; intervention group: n=121)	53 (43.1)	95 (78.5)
**Functional improvement, n (%)**			
	3 weeks (nonparticipant group: n=196; intervention group: n=150)	77 (39.3)	105 (56.5)
	6 weeks (nonparticipant group: n=130; intervention group: n=115)	66 (51.6)	91 (67.9)
	12 weeks (nonparticipant group: n=123; intervention group: n=121)	62 (50.8)	94 (77.7)

### Main Results

**Figure 2:**
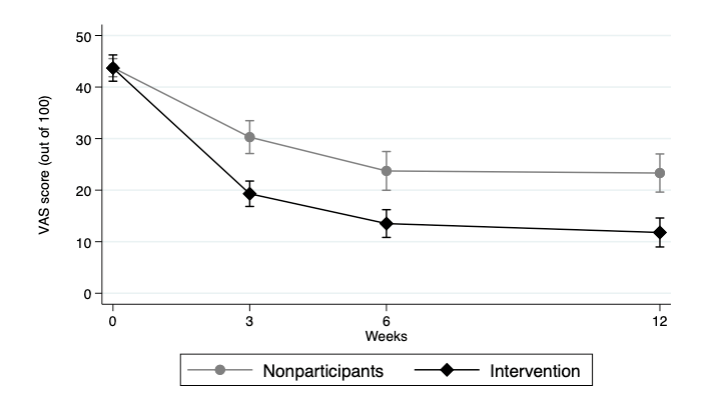
Adjusted VAS score over time. Results adjusted for age, pain region, registration month, health care service use, and time as fixed effects. VAS: visual analog scale.

The intervention group showed significantly lower adjusted pain scores at follow-up compared to nonparticipants (Figure 2). For nonparticipants, adjusted pain scores decreased from 43.8 (95% CI 42-45.5) at baseline to 30.3 (95% CI 27.1-33.5) at 3 weeks to 23.7 (95% CI 20-27.5) at 6 weeks to 23.3 (95% CI 19.6-27) at 12 weeks. For the intervention group, adjusted pain scores decreased from 43.7 (95% CI 41.1-46.2) at baseline to 19.3 (95% CI 16.8-21.8) at 3 weeks to 13.5 (95% CI 10.8-16.2) at 6 weeks to 11.8 (95% CI 9-14.6) at 12 weeks.

**Figure 3:**
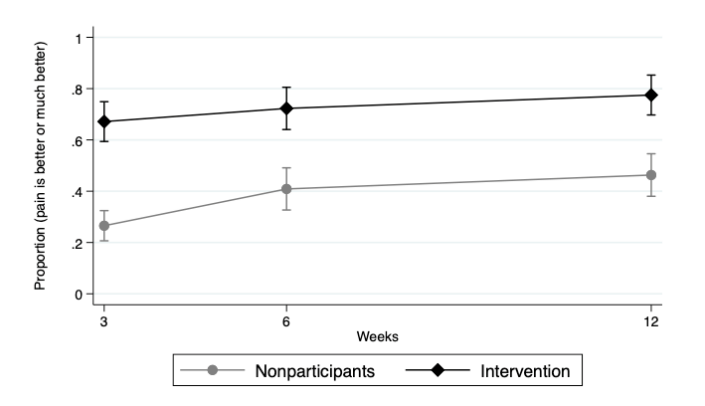
Adjusted proportion of participants reporting pain is better or much better over time. Results adjusted for age, baseline pain, pain region, registration month, health care service use, and time as fixed effects.

After adjustments, the intervention group showed a significantly higher percentage of people reporting pain was better or much better (PGIC) at follow-up versus nonparticipants. The adjusted percentage of nonparticipants who reported better or much better pain increased from 26.5% (95% CI 20.7%-32.4%) at 3 weeks to 40.9% (95% CI 32.7%-49.1%) at 6 weeks to 46.3% (95% CI 38%-54.6%) at 12 weeks. The adjusted percentage of intervention group who reported better or much better pain increased from 67.1% (95% CI 59.4%-74.9%) at 3 weeks to 72.3% (95% CI 64.1%-80.5%) at 6 weeks to 77.5% (95% CI 69.7%-85.3%) at 12 weeks (Figure 3).

**Figure 4:**
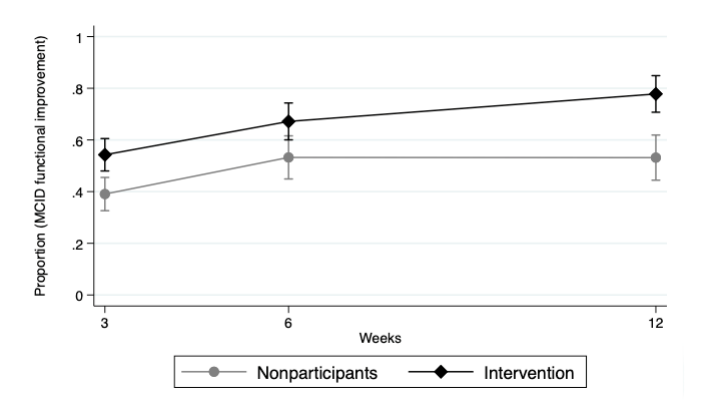
Adjusted proportion of participants with MCID in functional improvement over time. Results adjusted for age, baseline pain, pain region, registration month, health care service use, and time as fixed effects. MCID: minimal clinically important difference.

The intervention group showed a significantly higher percentage of people reporting functional improvement at 3 weeks and 12 weeks compared to nonparticipants. The adjusted percentage of nonparticipants reporting functional improvement increased from 39.1% (95% CI 32.6%-45.5%) at 3 weeks to 53.2% (95% CI 44.9%-61.6%) at 6 weeks to 53.2% (95% CI 44.4%-61.9%) at 12 weeks. The adjusted percentage of intervention group reporting functional improvement increased from 54.3% (95% CI 48%-60.5%) at 3 weeks to 67.2% (95% CI 60%-74.3%) at 6 weeks to 77.8% (95% CI 70.7%-84.9%) at 12 weeks (Figure 4).

Multimedia Appendix 1 shows the unadjusted and adjusted regression model results.

### Engagement

By 12 weeks, the intervention group averaged 1.8 (SD 1.1; range 1-6) video visits and 17.7 (SD 21.2; median 10; range 0-103) exercise therapy sessions.

## Discussion

### Principal Results and Generalizability

This observational study examined pain and function at 3, 6, and 12 weeks after starting a digital MSK program for acute and subacute MSK conditions versus nonparticipants. We found significant associations between the intervention and both pain improvement and PGIC at 3, 6, and 12 weeks. A significantly larger percentage of the intervention group also reported clinically meaningful functional improvement versus the nonparticipant group at 3 and 12 weeks.

As an observational study, we propose that findings are generalizable to the population of people with acute and subacute MSK pain with expressed interest in a digital acute MSK program. However, the study may not be generalizable to later adopters of health technology or all people with MSK pain.

### Comparison to Prior Work

VAS pain scores improved from baseline to follow-up for nonparticipants and intervention group members. However, the magnitude of pain improvement was significantly greater for the intervention group. The intervention group’s pain score improved from baseline by more than 10.9 points at 3 weeks, 10.1 points at 6 weeks, and 11.5 points at 12 weeks versus nonparticipants. This 10.1 to 11.5 point difference is similar to pain improvement shown in meta-analyses of spinal manipulative therapy (mean difference: 10; 95% CI 4-16) and exceeds that of nonsteroidal anti-inflammatory drugs for acute back pain (mean difference: 7; 95% CI 4-11) [27,28]. Our results are also consistent with recent meta-analyses reporting that exercise is an efficacious treatment for acute and subacute low back pain in the immediate term [9].

We detected statistically significant associations between the digital MSK program and meaningful functional improvement. In contrast, the effect of traditional services and medications on functional improvement have not been consistently demonstrated in acute MSK injuries [5]. Our study found that a significantly greater percentage of the intervention group reported meaningful functional improvement versus nonparticipants at 3 and 12 weeks, but not at 6 weeks. This may be due to the small sample size. We also suggest that nonparticipants’ function improved over time but at a slower rate than the intervention group. Furthermore, the intervention group continued to make progress in function beyond the 6-week mark, whereas nonparticipants’ functional improvement plateaued between 6 and 12 weeks. The ways that a digital acute MSK program changes the trajectory of functional improvement over time and in the long term are an area for additional research in the future.

We found that the intervention group averaged 1.8 video visits and 17.7 exercise therapy sessions by week 12. Although we did not collect self-reported information about exercises conducted without the app, this engagement data about completed exercise sessions demonstrated the feasibility of using app-based data to monitor member adherence to recommended exercises. This objective measure of adherence may supplement self-reports about efficacy and confidence in doing exercises. Adherence to exercises delivered through digital health programs has been shown to match or exceed that of in-person programs, and improved adherence is associated with better treatment outcomes for MSK needs [29-32].

### Strengths and Limitations

Study strengths include the use of data from 2 prospective cohorts who were similar in age, pain, and pain region at baseline. As a result, the study resulted in the longitudinal monitoring of a digital acute MSK program versus a nonparticipant group. Further, to our knowledge, our study is the first to evaluate a digital MSK program for acute and subacute needs against a nonparticipant group. The comparison group is essential given the natural history of acute and subacute MSK conditions. Improvement was assessed using 3 different outcomes, and we evaluated the program in real-world settings.

First, a study limitation is that this observational study cannot establish the causality of the intervention’s effect on outcomes. Second, we may have omitted important confounding variables (eg, motivation) that attenuate outcome estimates. Furthermore, we did not document the types of medications that study participants took to address pain and function. To build on current findings, we recommend a randomized controlled trial to establish causality and account for the effect of unmeasured factors. Third, more granular follow-up timepoints (eg, weekly) could provide more insight into the longitudinal course of pain and function in an acute digital MSK program. Future studies could use daily diaries to document exercise adherence and changes in daily pain to show time to pain resolution in days or weeks. Fourth, the study examines acute and subacute needs as a whole, and we do not report on outcomes for each region (ie, back, knee, shoulder, hip, or neck) separately. It is possible that the outcomes vary from region to region, and positive outcomes in one region might mask neutral or even negative outcomes in another region. To address this concern, we controlled for region in the regression models. Future studies could examine outcomes for specific regions or present stratified results.

### Conclusions

This study provided evidence that a digital acute MSK program may help improve pain and function in the short term among those with acute and subacute MSK needs. Future studies can build upon these results to further evaluate the extent to which digital health effectively manages a range of MSK needs, including acute and subacute needs.

## Appendix

Multimedia Appendix 1 Unadjusted and adjusted models comparing the intervention group to nonparticipants for each outcome.

## Abbreviations

HOOS-PS: Hip disability and Osteoarthritis Outcome Score Physical Function Short form
KOOS-PS: Knee injury and Osteoarthritis Outcome Score Physical Function Short form
MCID: minimal clinically important difference
MSK: musculoskeletal
PGIC: patient’s global impression of change
PT: physical therapist
RMDQ-11: 11-item Roland Morris Disability Questionnaire
sf-NPAD: Neck Pain and Disability Scale short form
SPADI: Shoulder Pain and Disability Index
VAS: visual analog scale

## Data Availability

The data sets generated during and/or analyzed during the current study are not publicly available because data are proprietary but are available from the corresponding author on reasonable request.
