# Supplementary material for: Clinical Outcomes After a Digital Musculoskeletal Program for Acute and Subacute Pain: Observational, Longitudinal Study With Comparison Group
Source: JMIR Rehabil Assist Technol. 2022 Jun 27;9(2):e38214. doi: 10.2196/38214 (PMC9274396; doi:10.2196/38214)
Supplement: Multimedia Appendix 1 [file rehab_v9i2e38214_app1.docx]

# Multimedia appendix 1. Unadjusted and adjusted models comparing the intervention group to nonparticipants for each outcome

## Pain

|  | Unadjusted | | | Adjusted | | |
| --- | --- | --- | --- | --- | --- | --- |
|  | Coefficient | 95% CI |  | Coefficient | 95% CI |  |
| intervention | 0.3 | -2.9 | 3.4 | -0.1 | -3.2 | 3.0 |
|  |  |  |  |  |  |  |
| weeks |  |  |  |  |  |  |
| 3 | -11.5 | -14.8 | -8.2 | -13.5 | -16.9 | -10.1 |
| 6 | -17.9 | -21.9 | -14.0 | -20.0 | -24.1 | -16.0 |
| 12 | -18.2 | -22.1 | -14.3 | -20.4 | -24.5 | -16.4 |
|  |  |  |  |  |  |  |
| acute#weeks |  |  |  |  |  |  |
| intervention * 3 | -12.5 | -17.1 | -7.9 | -10.9 | -15.6 | -6.2 |
| intervention * 6 | -11.1 | -16.4 | -5.8 | -10.1 | -15.5 | -4.8 |
| intervention * 12 | -12.3 | -17.6 | -7.0 | -11.5 | -16.9 | -6.1 |
|  |  |  |  |  |  |  |
|  |  |  |  | Results adjusted for age, pain region, registration month, health care service use, and time as fixed effects | | |

##

## PGIC

|  | Unadjusted | | | Adjusted | | |
| --- | --- | --- | --- | --- | --- | --- |
|  | Odds ratio | 95% CI |  | Odds ratio | 95% CI |  |
| intervention | 6.4 | 4.0 | 10.3 | 6.4 | 4.0 | 10.6 |
|  |  |  |  |  |  |  |
| weeks |  |  |  |  |  |  |
| 6 | 1.8 | 1.2 | 2.6 | 2.0 | 1.3 | 3.0 |
| 12 | 2.2 | 1.4 | 3.3 | 2.5 | 1.6 | 4.0 |
|  |  |  |  |  |  |  |
| acute#weeks |  |  |  |  |  |  |
| intervention * 6 | 0.7 | 0.4 | 1.2 | 0.6 | 0.4 | 1.2 |
| intervention * 12 | 0.8 | 0.4 | 1.4 | 0.7 | 0.3 | 1.4 |
|  |  |  |  |  |  |  |
|  |  |  |  | Results adjusted for age, baseline pain, pain region, registration month, health care service use, and time as fixed effects | | |

##

## Functional improvement

|  | Unadjusted | | | Adjusted | | |
| --- | --- | --- | --- | --- | --- | --- |
|  | Odds ratio | 95% CI |  | Odds ratio | 95% CI |  |
| intervention | 2.0 | 1.3 | 3.0 | 2.1 | 1.3 | 3.2 |
|  |  |  |  |  |  |  |
| weeks |  |  |  |  |  |  |
| 6 | 1.6 | 1.2 | 2.3 | 2.0 | 1.3 | 3.0 |
| 12 | 1.6 | 1.1 | 2.3 | 2.0 | 1.3 | 3.0 |
|  |  |  |  |  |  |  |
| acute#weeks |  |  |  |  |  |  |
| intervention * 6 | 1.0 | 0.6 | 1.6 | 1.0 | 0.5 | 1.7 |
| intervention * 12 | 1.7 | 1.0 | 2.9 | 1.8 | 0.9 | 3.4 |
|  |  |  |  |  |  |  |
|  |  |  |  | Results adjusted for age, baseline pain, pain region, registration month, health care service use, and time as fixed effects | | |

#

# 
